# DPOFA, a Cl^-^/HCO_3_^- ^exchanger antagonist, stimulates fluid absorption across basolateral surface of the retinal pigment epithelium

**DOI:** 10.1186/1471-2415-11-33

**Published:** 2011-11-15

**Authors:** Pavel Iserovich, Qiong Qin, Konstantin Petrukhin

**Affiliations:** 1 Department of Ophthalmology, Columbia University Medical Center, New York, NY, USA

## Abstract

**Background:**

Retinal detachment is a disorder of the eye in which sensory retina separates from the retinal pigment epithelium (RPE) due to accumulation of fluid in subretinal space. Pharmacological stimulation of fluid reabsorption from subretinal space to choroid across the RPE has been suggested as a treatment strategy for retinal detachment. DPOFA, (R)-(+)-(5,6-dichloro 2,3,9,9a-tetrahydro 3-oxo-9a-propyl-1H-fluoren-7-yl)oxy]acetic acid, is an abandoned drug capable of inhibiting Cl^-^/HCO_3_^- ^exchanger activity. We hypothesized that DPOFA may increase fluid absorption across basolateral surface of the RPE.

**Methods:**

Reverse transcription polymerase chain reaction (RT-PCR) analysis of mRNA for six different transporters that may act as Cl^-^/HCO_3_^- ^exchangers was conducted in bovine and human RPE to confirm that RPE from two species expresses the same repertoire of Cl^-^/HCO_3_^- ^exchanger isoforms. The degree of amino acid homology between orthologous human and bovine RPE-specific isoforms was calculated after performing protein alignments. Transport of fluid across bovine RPE-choroid explants mounted in the Ussing chamber was used to assess the ability of DPOFA to modulate fluid absorption across the RPE.

**Results:**

Using RT-PCR we showed that three isoforms (SLC4A2, SLC4A3, and SLC26A6) are strongly expressed in human and bovine RPE preparations. Amino acid comparisons conducted for RPE-specific isoforms support the use of bovine RPE-choroid explants as an adequate experimental system for assessing fluid absorption activity for DPOFA. Our data is consistent with the fact that DPOFA stimulates fluid absorption across the RPE in bovine RPE-choroid explants.

**Conclusions:**

DPOFA seems to stimulate transport of water across the RPE in bovine RPE-choroid explants. Additional experiments are required to establish dose-dependent effect of DPOFA on fluid absorption in the bovine RPE-choroid experimental system.

## Background

Retinal detachment (RD) is the most common cause of blindness in young adults [1-3]. In RD neuro-sensory retina separates from the underlying pigment epithelium due to accumulation of fluid in the subretinal space [4]. The only therapy for RD is surgical re-attachment. Surgery is most effective only if performed within 1-3 days after the disease onset. The rate of complications, often in a form of retinal re-detachment, is 10-20%, even if successful surgical re-attachment is performed in time [1]. Even when anatomical recovery in the form of retina reattachment is successfully accomplished, functional recovery after surgery may be poor due to the loss of photoreceptor cells. Identification of the pharmacological treatment for RD that can be used as adjunctive therapy to improve functional outcomes following surgery and reduce the rate of post-operative complications is of upmost importance. Pharmacological up-regulation of fluid reabsorption from subretinal space to choroid across the retinal pigment epithelium (RPE) has been suggested as potential treatment strategy for retinal detachment [5]. In recent years, several drug candidates have been tested *in vivo *and *in vitro *for the ability to stimulate subretinal fluid resorption [6-11]. However, identification of clinically proven pharmacological therapy capable of increasing reabsorption of subretinal fluid in retinal detachment patients remains enigmatic. Removal of fluid from subretinal space across the RPE is mainly driven by transport of K^+ ^and Cl^- ^[12,13]. As basolateral Cl^-^/HCO_3_^-^exchanger recycles Cl^- ^back to the RPE thus reducing the rate of fluid absorption from subretinal space, the net movement of water out of the RPE across basolateral surface is determined by activity of the Cl^-^/HCO_3_^- ^antiporter [14]. Inhibition of the Cl^-^/HCO_3_^-^exchanger would predictably lead to increase in water transport across the RPE. DPOFA, (R)-(+)-(5,6-dichloro 2,3,9,9a-tetrahydro 3-oxo-9a-propyl-1H-fluoren-7-yl)oxy]acetic acid, is an abandoned fluorenone drug that has been systemically administered to humans in clinical trials for trauma-induced brain damage [15-17]. While the primary molecular target for DPOFA is thought to be a Cl^-^/HCO_3_^- ^exchanger [18-20], a Cl^- ^channel blocker activity has also been suggested for this drug [21]. In the present study we conducted preliminary analysis of the effect of DPOFA on fluid transport using the bovine choroid-RPE *ex vivo *system.

## Methods

### DPOFA synthesis

**Figure 1:**
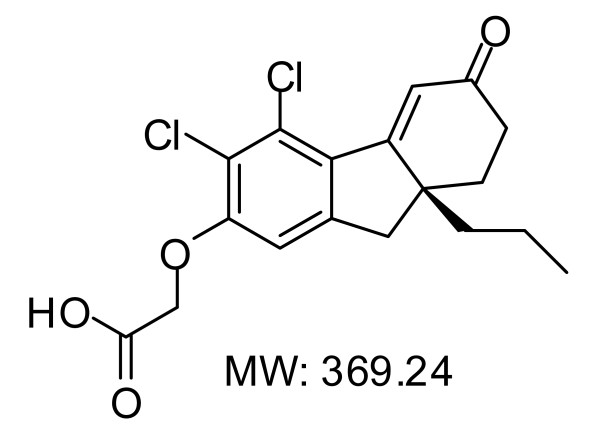
**Chemical structure of DPOFA**. The compound is also known in the literature as B-3(+) [20] and L-644,711 [18].

Chemical structure of DPOFA is shown in Figure 1. DPOFA is not commercially available. A non-GMP batch of DPOFA was synthesized by GVK Biosciences, Hyderabad, India. HPLC purity of the synthesized compound was confirmed and estimated to be 98%. Data from the ^1^H NMR (400 MHz, CDCl_3_) and mass spectrometry analyses were in agreement with the compound structure.

### Preparation of solutions

DPOFA's stock solution (40 mM) was prepared in 4.2% NaHCO_3_, pH 6.3 as described previously [22,23]. The medium used in fluid transport experiments was HEPES-HCO_3_^-^Ringer solution containing (in mM) 94.1 NaCl, 37 NaHCO_3_, 3.8 KCl, 1 KH_2_PO_4_, 0.8 MgSO_4_, 1.7 CaCl_2_, 6.9 glucose, and 20 HEPES. The pH was 7.4, and the osmolarity was 290 mosmol/kgH_2_O. The solution was pre-incubated at 37°C in 95%/5% air/CO_2 _for at least 16 hours before experiments.

### Preparation of RPE-choroid explants

Bovine eyes were obtained from Smithfield Beef Group, Souderton, PA and kept in Ringer's solution until their use within 2.5-5 hours after enucleation. The eye was dissected posteriorly to the ora serrata; lens and vitreous were removed. Neurosensory retina was peeled away; a circular area containing the RPE and choroid was cut out using a cork bore. The RPE-choroid tissue preparation was placed on a metal mesh disk choroid (basal) side down; nylon mesh was positioned on the RPE (apical) side. The tissue explant was inserted between two halves of the Ussing chamber which was pre-equilibrated at 37°C.

### Transport of fluid

The Ussing chamber was filled with pre-warmed Ringer's solution, and electric resistance was tested to assure tissue integrity. If electrical resistance was less than 100Ω, the preparation was discarded. Two cylinders containing measuring capillaries were inserted into apical and basal reservoirs of the Ussing chamber. Levels of liquid in capillaries were adjusted using the specially designed long barrel syringe. DPOFA was added to both apical and basal reservoirs of the Ussing chamber. We used horizontal observation microscope with automatic position reading in order to measure changes in the position of a meniscus in the measuring capillaries in response to fluid transport from apical to basal reservoir. The pumping rate was recorded as an increase in a meniscus position of the basal capillary expressed in micrometers. SigmaStat 3.0.1 package for Windows from SPSS Inc. was used for data analysis.

### RPE cell culturing, isolation of RNA, and RT-PCR

Primary fetal human RPE cell culture [24] was obtained from Dr. Sheldon Miller, National Eye Institute. As stated in reference [24], the research on preparation of this fetal human RPE culture followed the tenets of the Declaration of Helsinki and was approved by the NIH institutional review board. Primary fetal human RPE cells were cultured according the published procedure [24]. Bovine RPE was gently brushed off of the eyecup after discarding the lens, vitreous, and neurosensory retina. Total RNA was isolated from human RPE cells and from bovine RPE tissue using the RNeasy kit (Qiagen) and a manufacturer's protocol. After oligo(dT)-primed cDNA synthesis, which was performed with the ProtoScript First Strand cDNA Synthesis kit (New England Biolabs), we confirmed expression of the established RPE marker, bestrophin 1, using RT-PCR with the following bestrophin 1 primers: 5'-GCAGAACACAAGCAGTTGGA-3' and 5'-TCTCCAAAGGGGTTGATGAG-3' for human RPE; 5'-CAACCCATTTGGAGAGGATG-3' and 5'-AAGGTTCTTGGGCTGGTTCT-3' for bovine RPE. To assess expression of anion transporter isoforms we conducted RT-PCR analysis using the following combinations of oligonucleotide primers corresponding to different human and bovine transporters: human SLC4A1, 5'- TCTTCCAGGACCACCCACTA-3' and 5'-CATGCCTACTACCAGCAGCA-3'; human SLC4A2, 5'-CCAGTGGATTCTCGGTGACT-3' and 5'-ATCCCGTTAAGGGAGGTGAC-3'; human SLC4A3, 5'-CACCTACACGCAGAAGCTGA-3' and 5'- GGAAGATCCCAAAGAGCACA-3'; human SLC26A3, 5'-CCATCATCGTGCTGATTGTC-3' and 5'-GCCTTGCTTCTGCAGTTTTC-3'; human SLC26A4, 5'- GGGCTGGATCTCGGTTTACT-3' and 5'-CAAGGCTATGGATTGGCACT-3'; human SLC26A6, 5'-TTATCGGAGGCATCTTCCAG-3' and 5'- CAGCATTGGCAAAGTACACG-3'; bovine SLC4A1, 5'- TCTTCCAGGATCATCCACTGCA -3' and 5'-GCCTATGATCAGCAGCAGGT -3'; bovine SLC4A2, 5'- CCAGTGGCTTCTCAGTGACA -3' and 5'- ATCCCATTCAGGGAGGTCA -3'; bovine SLC4A3, 5'-CACCTACACACAGAAGCTGACG -3' and 5'- CAGGAAAATCCCAAAGAGCA -3'; bovine SLC26A3, 5'- CTATCATTGTGCTGATTGTCGTT -3' and 5'-GGCCTTTCTTCTGCAGTTTTC -3'; bovine SLC26A4, 5'-GGGACTGGACCTCGGTTTACT -3' and 5'- GGCTATGGATTGGCACTTTG -3'; bovine SLC26A6, 5'- GGGCATCTTCCAGTGCTTC -3' and 5'- GGCGTTGGCGAAGTACAC -3'. Fragment sizes for all Cl^-^/HCO_3_^- ^exchanger PCR products were within the 493-509 b.p. range. Control GAPDH primers were 5'-GGTCTTACTCCTTGGAGGCCATGT-3' and 5'- GACCCCTTCATTGACCTCAACTACA-3' for human cDNA (1 kb product size), and 5'-ATGGTGAAGGTCGGAGTGAA-3' and 5'-ATGCCAAAGTGGTCATGGAT-3' for bovine cDNA (503 b.p. product size). Primer3 program combined with BLAST search algorithm (Primer-BLAST available form NCBI at http://www.ncbi.nlm.nih.gov/tools/primer-blast/) was used to design all primers. Primer-BLAST allows for specificity check of the selected primers against the set of reference sequences. PCR was conducted according to the previously described touch-down protocol [25] with 26 cycles at annealing temperature of 55°C.

### Amino acid comparisons

Protein alignments were performed at the EMBL-EBI computer server using the ClustalW2 algorithm ( http://www.ebi.ac.uk/Tools/msa/clustalw2/). Amino acid sequence comparisons were conducted for the following currently available SLC4A2, SLC4A3, and SLC26A6 protein sequences (GenBank accession numbers are shown in parentheses): human SLC4A2 [GenBank:NP_001186621], human SLC4A3 [GenBank:NP_005061], human SLC26A6 [GenBank:NP_001035544], bovine SLC4A2 [GenBank:NP_001192593], bovine SLC4A3 [GenBank:XP_615029], bovine SLC26A6 [GenBank:NP_001070320], guinea pig SLC4A2 [GenBank:NP_001166488], rabbit SLC4A2 [GenBank:NP_001075788], and rabbit SLC4A3 [GenBank:NP_001075499].

## Results

**Figure 2:**
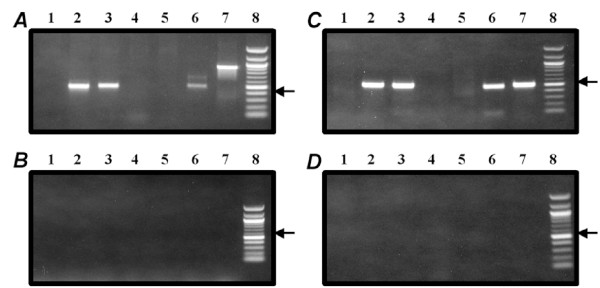
**Expression analysis of Cl**^-^**/HCO3**^- ^**exchanger isoforms in human and bovine RPE**. RT-PCR amplification was conducted on human (***A***) and bovine (***C***) RPE cDNA preparations. Panels ***B ***and ***D ***show the results of control PCR amplification with human (***B***) and bovine (***D***) isoform-specific primers performed with water in place of cDNA. Lanes 1-6 indicate PCR reactions performed with human (panels ***A***, ***B***) and bovine (panels ***C***, ***D***) oligonucleotides specific for SLC4A1, SLC4A2, SLC4A3, SLC26A3, SLC26A4, SLC26A6, respectively. Lane 7 represents amplification of the control GAPDH fragment performed with human (panels ***A***, ***B***) or bovine (panels ***C***, ***D***) oligonucleotide primers. Lane 8 contains size markers (100 b.p ladder) with arrow indicating a position of the 500 b.p. band.

**Figure 3:**
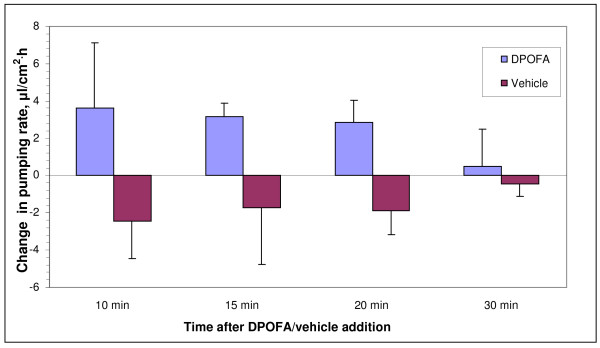
**Comparison of changes in water pumping rates in bovine RPE-choroid explants induced by DPOFA and vehicle treatment**. DPOFA at 1-20 μM concentrations was added to apical and basal baths of the chamber. The number of experiments and change in pumping rate values are shown in Table 1. Mean values are plotted with error bars depicting Standard Error of Means (SEM). The change in pumping rate from apical to basal side of the RPE-choroid explant was calculated at 10, 15, 20, and 30 minutes after drug and vehicle addition. While no statistically significant difference between compound doses could be discerned, we were able to detect the statistically significant (p = 0.013) increase in fluid absorption after drug treatment versus vehicle control during the first 20 minutes post drug/vehicle addition when the data for all DPOFA concentrations within the 1-20 μM were pooled for the analysis.

It has been reported that DPOFA shows significant species specificity in regard of inhibiting its main target, Cl^-^/HCO_3_^- ^exchanger. Human, cat, guinea pig, and rabbit constitute responsive species with similar high levels of DPOFA inhibitory activities [18,19,26], while rodents (rat and mouse) represent non-responsive species [20,27]. Before conducting experiments on fluid absorption in bovine RPE-choroid system we wanted to confirm that isoform specificity of Cl^-^/HCO_3_^- ^transporter in bovine RPE is similar to that of the human RPE cells, to assure that data obtained in bovine RPE system would be predictive of outcomes in the human retina. It has been reported that six different transporters from two protein families may act as Cl^-^/HCO_3_^- ^exchangers: SLC4A1, SLC4A2, SLC4A3, SLC26A3, SLC26A4, and SLC26A6 [28,29]. We purified total RNA from cultured primary fetal human RPE cells and from RPE tissue isolated from bovine eyes. Following cDNA synthesis from human and bovine RNA, we confirmed expression of the established RPE-specific marker, bestrophin 1, using RT-PCR (data not shown). We synthesized oligonucleotide primers from identical isogenic regions of six human and bovine Cl^-^/HCO_3_^- ^transporters and conducted RT-PCR analysis in order to compare isoform specificity of expressed anion transporters in human and bovine RPE. As shown in Figure 2, the repertoire of Cl^-^/HCO_3_^- ^exchanger isoforms expressed in the RPE is identical in human and *Bos taurus *with three isoforms (SLC4A2, SLC4A3, and SLC26A6) pronouncedly expressed in both RPE preparations. Control RT-PCR reactions conducted with water in place of cDNA confirmed specificity of PCR amplification (Figure 2, panels *B, D*). The degree of amino acid homology between orthologous human and bovine Cl^-^/HCO_3_^- ^isoforms expressed in the RPE was calculated after performing protein alignments using ClustalW2 algorithm. The bovine-human homology for SLC4A2 and SLC4A3 isoforms is above 95% which is in the range of amino acid similarity between RPE-specific human transporters and Cl^-^/HCO_3_^-^exchangers from three other "responsive" for DPOFA species, cat, guinea pig, and rabbit (data not shown). Lack of sequence information for the SLC26A6 transporter from cat, guinea pig, and rabbit precluded us from performing similar analysis for this isoform. Overall, amino acid comparisons conducted for SLC4A2 and SLC4A3 isoforms are consistent with the idea that *Bos taurus*, along with human, cat, guinea pig and rabbit may constitute a responsive for DPOFA species further supporting the use of bovine RPE-choroid explants as an adequate experimental system for assessing fluid absorption activity for DPOFA. Attempting to define a dose-dependent effect of the test compound on fluid absorption, we conducted a series of experiments comparing DPOFA concentrations in the 1-20 μM range with the effect of a compound vehicle over the period of 30 minutes. While no statistically significant difference between compound doses could be established, we were able to detect statistically significant increase in fluid absorption after drug treatment versus vehicle control during the first 20 minutes after drug or vehicle addition when data for all DPOFA concentrations were combined. When added in 1-20 μM concentrations, DPOFA significantly (p = 0.013) increased water absorption within first 20 minutes by 3.22 ± 1.4 μl/cm^2.^h while vehicle control decreased absorption by 2.1 ± 1.39 μl/cm^2.^h (Figure 3). Statistical significance could not be reached when 10, 15, and 20 minute timepoints were analyzed individually. The decrease in fluid absorption by the RPE in response to vehicle addition is reminiscent of the previously reported volume flow decline induced by "sham" treatment of choroid-RPE explants mounted in Ussing-type chambers [30]. Table 1 shows numeric data on changes in pumping rate over the period of 30 minutes in response to addition of 1-20 μM DPOFA or vehicle with the number of experiments indicated for each timepoint.

**Table 1:** Change in pumping rate in response to DPOFA or vehicle treatment

Time after addition, min.	**DPOFA, Change in pumping rate**^**1**^	**DPOFA, *SEM***^**2**^	**Vehicle, Change in pumping rate**^**1**^	**Vehicle, *SEM***^**2**^
10	3.64 (6)	3.49	-2.44 (5)	2.03
15	3.17 (4)	0.72	-1.71 (4)	3.08
20	2.83 (6)	1.22	-1.89 (4)	1.30
30	0.47 (10)	2.04	-0.44 (7)	0.68

^**1 **^Change in pumping rate in comparison with time 0 when DPOFA or vehicle were added is expressed in (μl/cm^2.^h); ^**2**^SEM, Standard Error of Means; Number of experiments is shown in parenthesis.

## Discussion

Despite significant unmet medical need, there is no pharmacological therapy that was approved by regulatory agencies for treatment of retinal detachment. Pharmacological up-regulation of fluid reabsorption from subretinal space to choroid across the retinal pigment epithelium has been suggested as potential treatment strategy for retinal detachment [5]. Basolateral Cl^-^/HCO_3_^- ^exchanger in the retinal pigment epithelium is a reasonable drug target for up-regulation of fluid reabsorption as it recycles Cl^- ^back to the RPE thus reducing the rate of fluid absorption from subretinal space. DPOFA (shown in Figure 1), also known in the literature as B-3(+) [20] and L-644,711 [18], is a non-diuretic small molecule Cl^-^/HCO3^- ^exchanger antagonist that has been systemically administered to humans in clinical trials for trauma-induced brain damage [15-17]. It has been shown that DPOFA exhibits remarkable species specificity in regard of inhibiting its main target, Cl^-^/HCO_3_^- ^exchanger. While the IC_50 _of 2 × 10^-11 ^M in the K^+^-induced swelling assay is reported in one of the responsive species, cats, the IC_50 _in a similar rat assay is only 2 × 10^-7 ^M [16]. It has been shown that human, cat, guinea pig, and rabbit constitute responsive species with similar levels of DPOFA inhibitory activities [18,19,26], while rodents (rat and mouse) represent non-responsive species [20,27]. Bovine RPE-choroid system is a widely used experimental tool for assessing the effect of drug treatment on reabsorption of fluid across the RPE [31-33]. However, in light of significant species-specific difference in response to DPOFA in tissue slice-based CNS experimental systems we wanted to develop additional support for using bovine RPE-choroid system as a tool capable of predicting DPOFA response in human RPE. DPOFA's selectivity for different Cl^-^/HCO_3_^- ^exchanger isoforms is not known. Assuming that species-specific difference in response to DPOFA may be caused by species-specific variations in repertoire of expressed Cl^-^/HCO3^- ^transporters, we compared isoform specificity of Cl^-^/HCO_3_^- ^transporters expressed in human and bovine RPE. Using RT-PCR analysis conducted with isoform-specific oligonucleotide primers that were designed from the isogenic regions of human and bovine genes we showed that three isoforms (SLC4A2, SLC4A3, and SLC26A6) are strongly expressed in both RPE preparations (Figure 2). After defining transporters expressed in the RPE, we conducted their amino acid comparisons in order to confirm that human-bovine protein homology for RPE-specific transporters is in line with the degree of similarity between human and three other responsive species (cat, guinea pig, and rabbit). These protein comparisons confirmed that human-bovine homology for SLC4A2 and SLC4A3 does not differ from homology of RPE specific transporters in responsive for DPOFA species which may further indicate that effect of the drug in bovine RPE-choroid system is likely to adequately reflect the behavior of DPOFA in human RPE. In an attempt to define a dose-dependent effect of DPOFA on reabsorption of fluid in the bovine RPE-choroid system, we conducted multiple experiments assessing compound activity in the 1-20 μM dose range on stimulation of water absorption across the tissue explant (Table 1 and Figure 3). While no statistically significant difference between compound doses could be discerned, we were able to detect statistically significant (p = 0.013) increase in fluid absorption after drug treatment versus vehicle control during the first 20 minutes post drug/vehicle addition when data for all DPOFA concentrations within the 1-20 μM range were pooled for the analysis. We speculate that our inability to detect dose dependence in compound activity is consistent with reported complexity of biological response to DPOFA in another biological system. It has been shown that titrations of DPOFA in a cerebrocortical slice swelling assay produced the U-shaped dose response curve with a maximum reduction of K^+^-induced swelling in a nanomolar range followed by decrease of drug activity at higher micromolar concentrations [19,26]. Compound evaluation outside of the micromolar range is essential for establishing dose-dependent effect of DPOFA on fluid absorption. Compound re-synthesis and additional studies are required in order to elucidate detailed pharmacological mechanisms of DPOFA activity in the RPE-choroid explant system.

## Conclusion

Preliminary analysis of DPOFA, a non-diuretic small molecule Cl^-^/HCO_3_^- ^exchanger antagonist, indicates that this compound increases reabsorption of fluid across basolateral surface of the retinal pigment epithelium in the *ex vivo *bovine RPE-choroid system. Our results provide rationale for additional experimental evaluation of this compound as a potential treatment for retinal detachment.

## Abbreviations

DPOFA: (R)-(+)-(5,6-dichloro 2,3,9,9a-tetrahydro 3-oxo-9a-propyl-1H-fluoren-7-yl)oxy]acetic acid; RPE: retinal pigment epithelium; HEPES: (4-(2-hydroxyethyl)-1-piperazineethanesulfonic acid).

## Competing interests

Columbia Technology Ventures, the technology transfer branch of Columbia University, filed a provisional patent application covering the use of DPOFA for treatment of retinal detachment.

## Authors' contributions

PI conducted fluid transport experiments in bovine RPE-choroid and analyzed fluid absorption data; QQ performed RT-PCR experiments; KP selected the compound and arranged for its synthesis, participated in the analysis of the fluid absorption data, analyzed RT-PCR results, performed amino acid sequence comparisons, and drafted the manuscript. All authors read and approved the final manuscript.

## Pre-publication history

The pre-publication history for this paper can be accessed here:

http://www.biomedcentral.com/1471-2415/11/33/prepub
